# Retinal malperfusion in albuminuric Type 1 diabetes mellitus patients without clinical signs of diabetic retinopathy: a prospective pilot study

**DOI:** 10.1186/s40942-017-0102-y

**Published:** 2017-12-18

**Authors:** Fernando Korn Malerbi, Caio Vinicius Regatieri, João Roberto de Sa, Paulo Henrique Morales, Michel Eid Farah, Sergio Atala Dib

**Affiliations:** 1 Department of Ophthalmology, Federal University of São Paulo, Av Divino Salvador, 398/41, São Paulo, SP 04078-011 Brazil; 2 Department of Endocrinology, Federal University of São Paulo, São Paulo, Brazil

**Keywords:** Type 1 diabetes mellitus, Diabetic retinopathy, Fluorescein angiography, Nephropathy, Albuminuria, Estimated glomerular filtration rate

## Abstract

**Background:**

To report fluorescein angiography findings in a group of albuminuric Type 1 diabetes mellitus (T1DM) patients without diabetic retinopathy.

**Methods:**

Fifteen albuminuric T1DM patients with normal/near normal estimated glomerular filtration rate without diabetic retinopathy underwent fluorescein angiography; presence of microaneurysms, vascular permeability changes and retinal malperfusion were evaluated.

**Results:**

Fluorescein angiography revealed microaneurysms, blood-retinal barrier breakdown and retinal ischemia in 10 (67%) and 11 (73%); 8 (53%) and 9 (60%); 2 (13%) and 5 (33%) of patients at baseline and follow up, respectively. Follow up time averaged 24.6 months, minimum follow up time was 20 months. Patients who presented retinal malperfusion had higher HbA1C and lower estimated glomerular filtration rate.

**Conclusions:**

Most albuminuric T1DM patients with a normal fundus exam had angiographic signs of diabetic retinopathy, some presenting retinal malperfusion. Retinal changes may be found with more sensitive testing in these patients, especially with impaired estimated glomerular filtration rate, even if the fundus exam is normal, and fluorescein angiography should be considered. These findings point to a homogenous presentation of the diabetic microangiopathies.

**Electronic supplementary material:**

The online version of this article (10.1186/s40942-017-0102-y) contains supplementary material, which is available to authorized users.

## Background

Diabetic retinopathy (DR) is the leading cause of visual impairment in developed countries [1]; visual acuity usually declines because of macular edema or retinal ischemia [2]. DR and diabetic nephropathy are microvascular complications of diabetes mellitus (DM) which share some similar risk factors and pathophysiology pathways, such as increased vascular permeability. These chronic diabetes complications may not arise simultaneously in Type 1 diabetes (T1DM) patients [3], but this heterogeneity might be due to inaccurate diagnosis [4].

It is a general consensus that DR should be annually screened if the fundus is normal [5, 6]. Retinal fluorescein angiography (FA), an exam that evaluates retinal perfusion, may detect preclinical diabetic retinopathy [7], such as retinal vessel permeability changes, microvascular leakage and blood-retina barrier breakdown. FA is not routinely performed in patients with a normal fundus exam.

Albuminuria is considered an important early marker of diabetic nephropathy and endothelial dysfunction [8], along with estimated glomerular filtration rate (eGFR). Patients already with diabetic nephropathy may be at a higher risk of developing other microvascular complications, such as retinopathy [5]. However, there are causes other than diabetes for renal impairment in diabetes patients, and these other renal diseases are present in approximately 30% of diabetic patients with macroalbuminuria without diabetic retinopathy; the absence of retinopathy is considered an indication for renal biopsy in albuminuric patients [9].

In order to evaluate retinal permeability and/or perfusion changes in albuminuric T1DM patients without clinical signs of diabetic retinopathy, we performed a clinical and angiographic follow up of such patients for a period of 2 years.

## Patients and methods

Inclusion criteria were T1DM, diagnosed in accordance with the American Diabetes Association criteria [10]; spot urinary albumin excretion rate between 30 and 300 mg/g creatinine, detected in at least two out of three samples [8], or use of angiotensin converting enzyme inhibitors or angiotensin receptor blocker secondary to previous diagnosis of albuminuria; and absence of clinical diabetic retinopathy. All patients were being followed in a University Diabetes Center. Exclusion criteria were spherical equivalent > ± 3 diopters, other ocular comorbidities that could interfere with the normal retinal aspect, such as vascular occlusions, inherited retinal diseases, as well as uveitis and media opacities that precluded retinal evaluation such as cataracts. Written informed consent was obtained from all subjects, and the local ethics committee approved the study protocol.

Clinical and laboratorial parameters such as age, gender, diabetes duration, arterial hypertension, estimated glomerular filtration rate (eGFR), lipid disorders and HbA1c values were obtained from the Diabetes Center clinical files.

HbA1c was measured by HPLC (TOSOH G7, Luxembourg, Belgium; nv: 4.0–5.6%). HbA1c values correspond to the arithmetic mean of available HbA1c measurements in the 2 years preceding the study visit. Urinary albumin from spot urine collections was determined using an immunoturbidimetric assay (nv: < 30 mg/g creatinine); eGFR was calculated with Chronic Kidney Disease Epidemiology Collaboration (CKD-EPI) equations (nv > 90 ml/min/1.73 m^2^) [11].

In order to evaluate the presence or absence of diabetic retinopathy, all included patients underwent clinical ophthalmological evaluation, which consisted of mydriatic binocular indirect ophthalmoscopy (BIO) with a 20 diopter condensing lens and slit lamp fundus biomicroscopy with a 78 diopter condensing lens. Retinal evaluation was performed by an experienced retinal specialist. All included patients had a normal fundus at baseline. After inclusion in the study, FA and optical coherence tomography (OCT) were performed.

### Fluorescein angiogram (FA)

FA was obtained after 2 ml intravenous injection of 25% fluorescein sodium. Retinal images were obtained with Heidelberg HRA2 (Heidelberg Engineering, Germany). Image analysis was performed with HRA software. FA analysis was also evaluated blindly by two independent experienced readers (FKM and CVR) and the following parameters were noted: retinal ischemia/malperfusion, microaneurysms, vascular changes (telangiectasis, vessel wall staining, vascular hyperpermeability), angiographic macular edema, retinal neovascularization, intraretinal microvascular abnormalities (IRMA). Blood-retinal barrier breakdown was defined as increased retinal fluorescein leakage associated with vascular changes [1], and retinal malperfusion was defined as the presence of avascular areas which could be detected because of the sharp contrast to the adjacent clearly visible capillary net [12]. Analyses were performed in both eyes of each subject, and the posterior pole was evaluated as well as retinal periphery. Early/midphase angiograms allowed evaluation of the foveal avascular zone, capillary loss and vascular changes, while late phase angiograms allowed evaluation of fluorescein leakage and cystoid changes [12].

### Spectral-domain optical coherence tomography (SD-OCT)

SD-OCT scans were obtained with spectral-domain Cirrus OCT (Carl Zeiss Meditec, Jena, Germany) and analysis was based on the automated central retinal thickness (CRT) protocol. Central retinal thickness was automatically measured in the Macular Cube protocol.

All patients were followed for a minimum of 20 months. According to the current guidelines [10], if the fundus was normal, they were asked to be seen again in 1 year, in an interim evaluation which consisted of BIO and slit lamp fundus biomicroscopy. At the 2 year visit, FA and OCT were repeated.

### Statistical analysis

Data are expressed as mean ± standard deviation. Analyses consisted of descriptive statistics and two-tailed Student’s *T* test for independent samples, performed after Levene’s test for equality of variances. A 95% confidence interval and a 5% level of significance were adopted. The following variables and outcomes from baseline and follow up were compared, respectively: HbA1C and eGFR; any FA changes or retinal malperfusion. All statistics were calculated using SPSS for Windows version 19.0 (Armonk, NY).

## Results

### Baseline evaluation

Clinical and laboratory data of the fifteen patients followed in this study are presented in Additional file 1: Table S1. Gender was feminine in 10 patients (67%). Two patients (13%) had systemic arterial hypertension and they were under control. Five patients (33%) were under treatment for lipid disorders with statins.

**Fig. 1:**
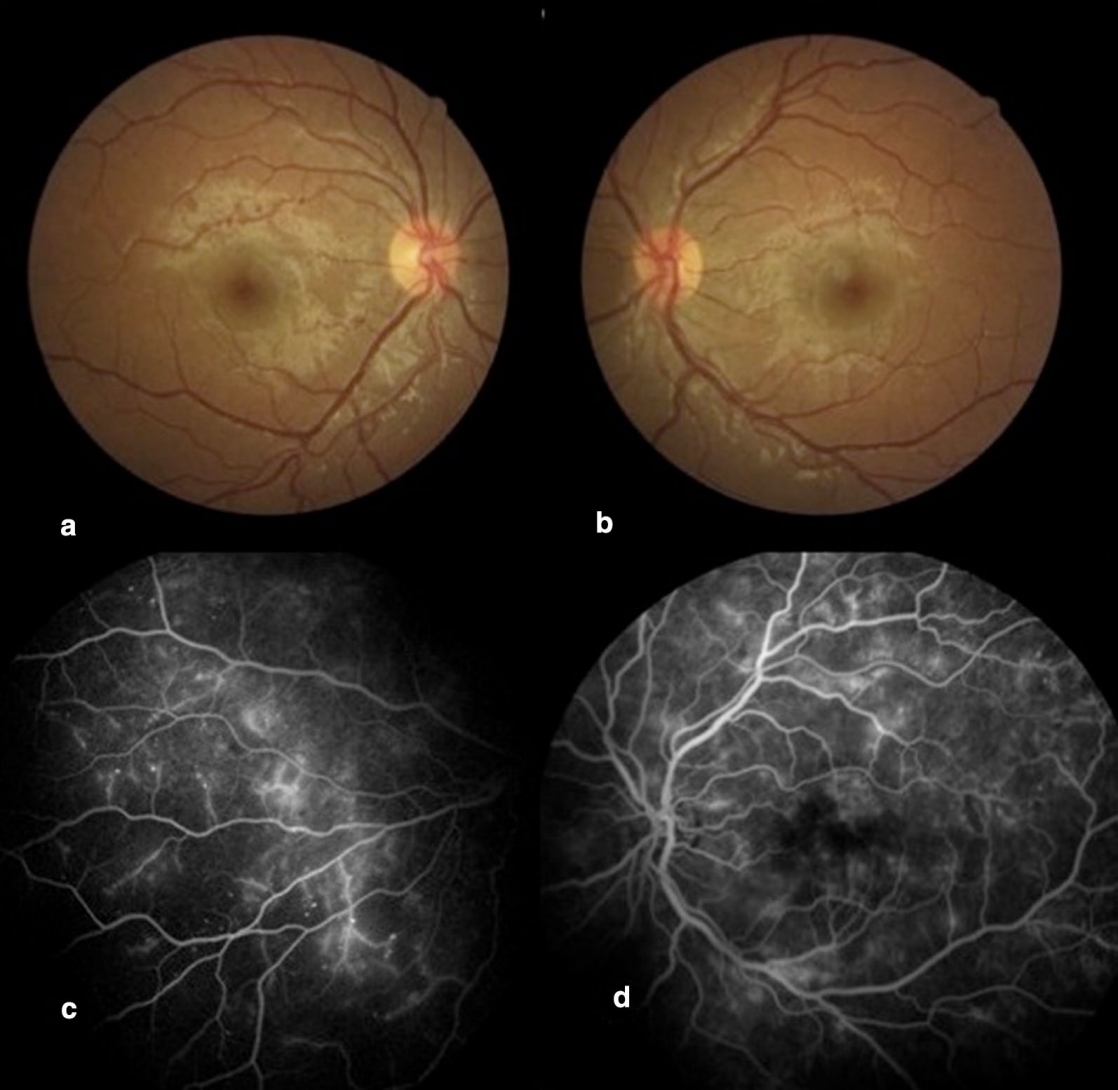
Fundus images and fluorescein angiogram of a microalbuminuric Type 1 DM patient. **a**, **b** Fundus photoghraphs depicting normal fundus (**a** right eye; **b** left eye). **c**, **d** Fluorescein angiogram frames of the same patient, depicting retinal ischemia (**c** right eye) and breakdown of blood-retinal barrier (**d** left eye)

At FA baseline analysis, ten patients (67%) had microaneurysms, blood-retinal barrier breakdown was evident in 8 (53%), and 2 (patients 6 and 13—Additional file 1: Table S1) (13%) showed signs of retinal malperfusion (Fig. 1); patients who presented retinal malperfusion had higher HbA1C levels than patients who did not present retinal malperfusion (13.2 ± 2.2 vs 9.3 ± 1.3%, p = 0.003). Agreement for FA analysis was complete between the two expert examiners.

SD-OCT scans were analyzed qualitatively for retinal architecture and quantitatively for retinal thickness. All eyes showed a normal anatomic pattern at SD-OCT analysis; no patient showed tomographic signs of macular edema. CRT was normal in all eyes (240 ± 18 μm).

### Interim evaluation

All patients presented with normal fundus except patient 6 (Additional file 1: Table S1) who developed retinal neovascularization and underwent laser pan retinal photocoagulation. This patient had baseline and follow up HbA1C values of 14.7 and 12.9%, respectively, and she was one of the two patients that had retinal malperfusion at the baseline evaluation.

### Follow up

Average follow up time was 24.6 months (range 20–32 months).

Additional file 1: Table S1 and Additional file 2: Graphic S1 show the evolution of clinical, laboratorial and FA parameters. CRT remained normal in all eyes (238 ± 22 μm).

One-third of the patients developed clinical signs of diabetic retinopathy at the end of follow up: 3 patients with mild nonproliferative DR, 1 moderate nonproliferative DR and 1 proliferative DR. Five patients who had FA abnormalities at baseline still had no clinically visible signs of DR at follow up.

Three patients showed stable FA findings, whereas 12 patients (80%) showed worsening of FA parameters; 3 of those patients did not have FA changes at baseline evaluation. A lower baseline eGFR was present in those patients who had FA changes as compared to patients who did not present FA changes at follow up (114 ± 25 vs 168 ± 55 ml/min/1.73 m^2^, p = 0.030). At follow up, five (33%) patients had retinal malperfusion, and they had higher baseline HbA1C levels (11.3 ± 2.1 vs 9.1 ± 1.3%, p = 0.024) and lower eGFR at follow up (88 ± 19 vs 115 ± 20 ml/min/1.73 m^2^, p = 0.026). All other comparisons failed to reach significance.

**Fig. 2:**
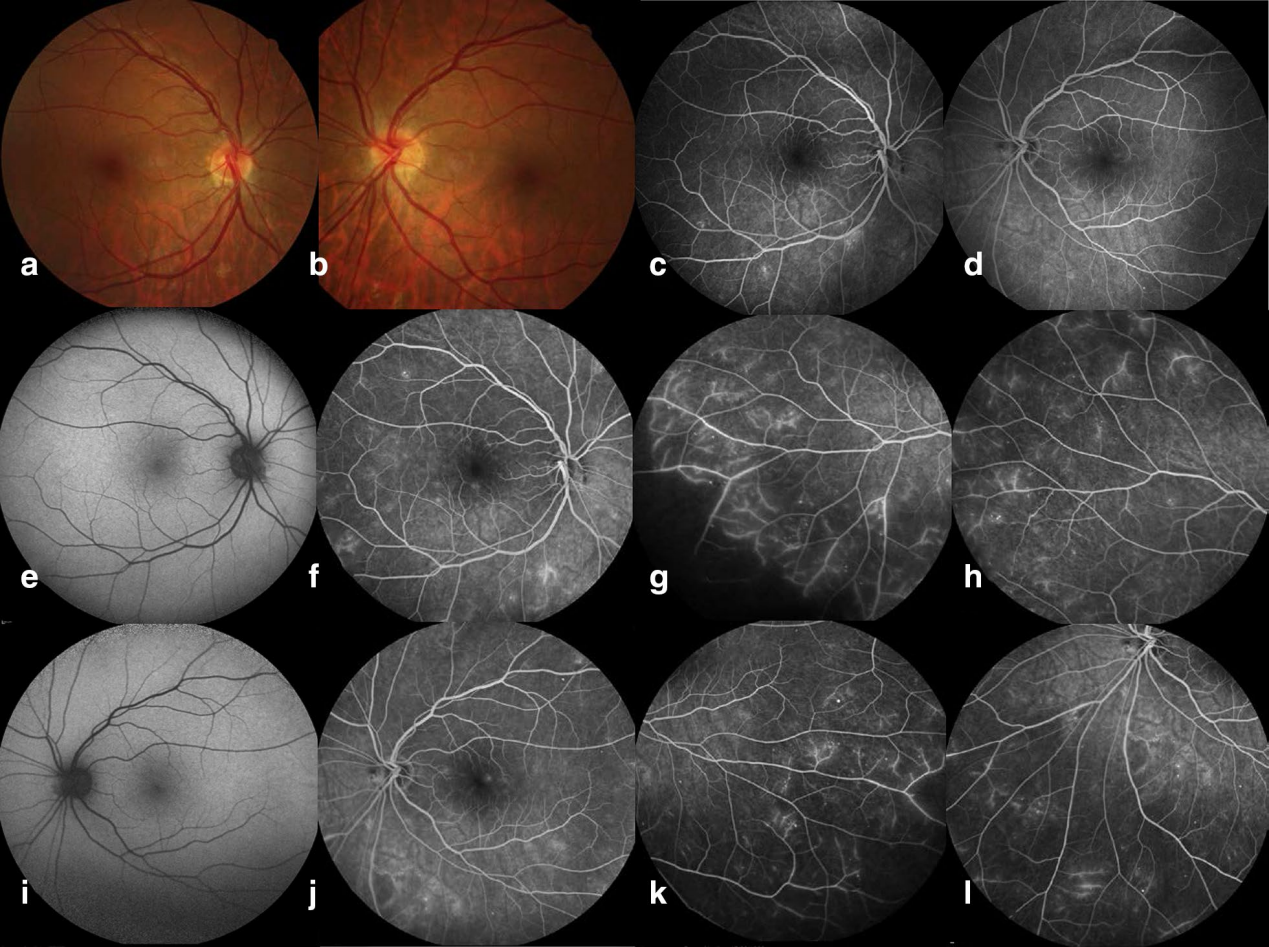
Baseline and follow up fundus and fluorescein angiogram of a microalbuminuric Type 1 DM patient. **a**, **b** Baseline color fundus photoghraphs depicting normal fundus (**a** right eye; **b** left eye). **c**, **d** Baseline fluorescein angiograms depicting leakage and macular cystoid changes. **e** Follow up fundus image of right eye depicting a single microaneurysm. **f**–**h** Follow up fluorescein angiograms of right eye depicting leakage, vessel wall staining, cystoid changes and peripheral retinal malperfusion. **i** Follow up fundus image of left eye depicting a normal fundus. **j**–**l** Follow up fluorescein angiograms of left eye depicting leakage, vessel wall staining, cystoid changes and peripheral retinal malperfusion

Eleven (73%) patients had microaneurysms at FA analysis, blood-retinal barrier breakdown was evident in 9 (60%), and 5 (33%) showed signs of retinal malperfusion or retinal neovascularization. Among the 5 patients who presented retinal malperfusion or retinal neovascularization at the follow up, two had already presented FA signs of retinal malperfusion at baseline (patients 6 and 13, Additional file 1: Table S1). Retinal malperfusion was detected on 3 patients who did not have FA signs of malperfusion at baseline (patients 5, 7 and 14, Additional file 1: Table S1). Two patients (patients 1 and 5, Additional file 1: Table S1) became pregnant during follow up period; one of them (patient 1) had a good metabolic control (HbA1C varied from 7.7 to 6.3%) and showed stable retinopathy (microaneurysms only), whereas the other (patient 5) had worsened metabolic control (HbA1C varied from 9.0 to 9.3%) and developed retinal malperfusion (Fig. 2).

In relation to other systemic alterations, two patients (patients 1 and 15, Additional file 1: Table S1) with systemic arterial hypertension were in the normal perfusion group; two other patients (patients 6, 14, Additional file 1: Table S1) under treatment for lipid disorders were in the malperfusion group and other three patients (patients 3, 8, 9, Additional file 1: Table S1) were in the normal perfusion group.

All eyes showed a normal anatomic pattern at SD-OCT analysis; no patient showed tomographic signs of macular edema. CRT was normal in all eyes (238 ± 23 μm).

## Discussion

In this pilot study, FA analysis detected that 67% of albuminuric T1DM patients without clinical signs of DR had at least one eye with angiographic signs of DR at baseline evaluation, and 13% of the patients had angiographic signs of retinal malperfusion. CRT was normal in all evaluated eyes.

After 2 years of follow-up, despite having been followed in a tertiary diabetes center, most patients had poor glycemic control (HbA1C ~ 9%), in accordance with published data regarding this age group [13]; 80% of these patients showed signs of clinical or angiographic DR worsening, and 33% of the patients had developed retinal malperfusion and/or retinal neovascularization. Patients who presented FA changes at follow up had a lower baseline eGFR.

Patients who presented retinal malperfusion at baseline and follow up had higher baseline HbA1C levels; patients who presented retinal malperfusion at follow up also had a lower eGFR at follow up.

The apparent dissociation between diabetic retinopathy and nephropathy has been described previously [3, 5]; however, with FA, which yields a more sensitive DR diagnostic testing than the clinical exam [1], the disparity between DR and nephropathy was reduced, as 86% of the patients in this study had FA signs of retinal disease at the end of follow up.

It is known that inflammation, endothelial damage and impaired regulation of extracellular matrix remodelling by matrix methaloproteinases are the background to both albuminuria and diabetic retinopathy [14, 15]; the latter and diabetic nephropathy also share similar risk factors and pathophysiology pathways. The results of the present study confirm these data and suggest that retinopathy should be actively sought once albuminuria and/or decreased eGFR are detected.

FA is a sensible method to diagnose preclinical diabetic retinopathy [1, 16] and it may detect increased retinal vascular permeability in pre-diabetic subjects, as well as in DM patients without clinical signs DR [1, 4, 17]. Our study is, to the best of our knowledge, the first one to detect FA signs of retinal malperfusion in T1DM patients without clinical signs of diabetic retinopathy. Retinal malperfusion may lead to severe and sometimes irreversible complications such as macular ischemia and retinal neovascularization [2].

It is well established that poor glycemic control is associated with the development of retinopathy and nephropathy in T1DM [18, 19]. Even though the belief that albuminuria precedes GFR loss has been recently challenged, both albuminuria and reduced GFR are considered hallmarks of renal damage in individuals with diabetes; they may be independent markers of chronic renal disease progression [20]. The present study contributes with the understanding that albuminuria plays a role as a marker of diabetic microvascular disease, since our albuminuric patients had also sub-clinical signs of diabetic retinopathy. This finding carries clinical importance: when one of the diabetic microvascular complications is present, the clinician should be careful regarding the appearance of the microvascular complications in other target organs. This search for complications should be emphasized, if possible with more sensitive tests, as was the case with fluorescein angiography.

This pilot study has some limitations. First, RD screening was not performed according to the gold-standard ETDRS protocol [21]. However, retinal examination with indirect ophthalmoscopy plus slit lamp fundus biomicroscopy is an accepted method for the screening of diabetic retinopathy [22] and represents the routine ophthalmic evaluation of diabetic patients in our country [23–26]. Second, control groups of normal individuals and normoalbuminuric T1DM patients for the comparison of FA findings were lacking. However, FA carries the potentially serious risk of anaphylaxis; furthermore, FA has already been extensively studied in normal and pre-diabetes individuals, as well as in DM patients without DR [1, 4, 17, 27, 28], all of whom have presented only hyperpermeability changes, in opposition to our findings of retinal malperfusion. Third, our series comprised a reduced number of patients. Nevertheless, even with a reduced sample, we found angiographic signs of retinal malperfusion in patients who would be screened as normals in the routine evaluation.

## Conclusions

In conclusion, our data suggest that dissociation between diabetic retinopathy and albuminuria is less frequent once more sensible DR testing is performed. Hence, albuminuric T1DM patients with a normal fundus should be carefully followed for retinal complications, preferably with FA. This pilot study also detected DR worsening in a relatively short period of time in a group of patients with poor glycemic control. Our conclusions warrant the need for further controlled studies to determine whether detection of preclinical DR by FA should prompt a demand for a customized follow up schedule with reduced screening intervals for this group of patients.

## Additional files

**Additional file 1: Table S1.** Baseline and Follow up Clinical and Laboratory Data of Type 1 Albuminuric Patients.

**Additional file 2: Graphic S1.** Clinical and angiographic evolution of albuminuric Type 1 Diabetes patients.

## Abbreviations

T1DM: Type 1 diabetes mellitus
DR: diabetic retinopathy
DM: diabetes mellitus
FA: fluorescein angiography
eGFR: estimated glomerular filtration rate
HbA1c: glycated hemoglobin
HPLC: high-performance liquid chromatography
CKD-EPI: Chronic Kidney Disease Epidemiology Collaboration
OCT: optical coherence tomography
SD-OCT: spectral-domain optical coherence tomography
CRT: central retinal thickness
ETDRS: Early Treatment Diabetic Retinopathy Study
