# Supplementary material for: Retinal malperfusion in albuminuric Type 1 diabetes mellitus patients without clinical signs of diabetic retinopathy: a prospective pilot study
Source: Int J Retina Vitreous. 2017 Dec 18;3:49. doi: 10.1186/s40942-017-0102-y (PMC5733928; doi:10.1186/s40942-017-0102-y)
Supplement: Supplementary file 1 — Additional file 1: Table S1. Baseline and Follow up Clinical and Laboratory Data of Type 1 Albuminuric Patients. [file 40942_2017_102_MOESM1_ESM.docx]

Table S1. Baseline and Follow up Clinical and Laboratory Data of Type 1 Albuminuric Patients

|  | BASELINE | | | | | | | FOLLOW UP | | | | |  |
| --- | --- | --- | --- | --- | --- | --- | --- | --- | --- | --- | --- | --- | --- |
| Patient number | Age  (yrs) | DM duration (yrs) | A1C  (%) | eGFR  (mL/min/1.73 m^2^) | Clin alt* | FA alt**¶** | FA MP **§** | A1C  (%) | eGFR  (mL/min/1.73 m^2^) | Clin alt* | FA alt**¶** | FA MP**§** | Evolution |
| 1 | 31 | 24 | 7.7 | 108.41 | - | + RE | - | 6.3 | 113,84 | - | +BE | - | stable, pregnancy |
| 2 | 12 | 5 | 8.97 | 129.43 | - | - | - | 8.5 | 138.61 | - | - | - | stable |
| 3 | 13 | 6 | 8.73 | 208.4 | - | - | - | 11.8 | 127.7 | - | - | - | stable |
| 4 | 20 | 11 | 11.45 | 122.28 | - | + BE | - | 11.9 | 111.74 | - | +BE | - | worse |
| 5 | 23 | 13 | 9 | 93.11 | - | + BE | - | 9.3 | 86.54 | + RE(MA) | +BE | +LE | worse, pregnancy |
| 6 | 19 | 11 | 14.75 | 148 | - | + BE | + BE | 12.9 | 64.55 | +BE (NV) | +BE | +BE | worse, PRP |
| 7 | 29 | 8 | 10.55 | 81.26 | - | + BE | - | 9 | 86.13 | +BE (MA) | +BE | + BE | worse |
| 8 | 28 | 21 | 9.8 | 154.84 | - | + BE | - | 8.4 | 83.55 | - | +BE | - | worse |
| 9 | 49 | 13 | 8.32 | 66.34 | - | + BE | - | 10.7 | 79.33 | - | +BE | - | worse |
| 10 | 14 | 3 | 10.62 | 125.44 | - | + LE | - | 10.2 | 126.49 | - | +BE | - | worse |
| 11 | 15 | 8 | 8.6 | 121 | - | - | - | 7 | 110.73 | - | + LE | - | worse |
| 12 | 17 | 11 | 7.23 | 113.88 | - | - | - | 8.9 | 123.38 | - | +LE | - | worse |
| 13 | 19 | 15 | 11.64 | 124.75 | - | + BE | + BE | 8.1 | 84.54 | +BE | +BE | +BE | worse |
| 14 | 28 | 8 | 10.8 | 96.73 | - | + BE | - | 10.5 | 117.95 | +RE | +BE | +LE | worse |
| 15 | 17 | 13 | 9.7 | 137.32 | - | - | - | 12.8 | 130.86 | - | +RE | - | worse |
| Mean | 22.2 | 11.3 | 9.8 | 122 |  |  |  | 9.7 | 106 |  |  |  |  |
| SD | 9.6 | 5.6 | 1.9 | 34 |  |  |  | 2.0 | 23 |  |  |  |  |

* Clinical Alteration; **¶** Fluorescein Angiography alteration; **§** Fluorescein Angiography Malperfusion

Abbreviations: RE (right eye), LE (left eye), BE (both eyes), MA (microaneurysm), NV (neovascularization), PRP (pan retinal photocoagulation)
