# Supplementary material for: Retinal malperfusion in albuminuric Type 1 diabetes mellitus patients without clinical signs of diabetic retinopathy: a prospective pilot study
Source: Int J Retina Vitreous. 2017 Dec 18;3:49. doi: 10.1186/s40942-017-0102-y (PMC5733928; doi:10.1186/s40942-017-0102-y)
Supplement: Supplementary file 2 — Additional file 2: Graphic S1. Clinical and angiographic evolution of albuminuric Type 1 Diabetes patients. [file 40942_2017_102_MOESM2_ESM.docx]

Graphic S1. Clinical and angiographic evolution of albuminuric Type 1 Diabetes patients.
